# Sex-related differences in length and erosion dynamics of human telomeres favor females

**DOI:** 10.18632/aging.100068

**Published:** 2009-07-14

**Authors:** Peter Möller, Susanne Mayer, Torsten Mattfeldt, Kathrin Müller, Peter Wiegand, Silke Brüderlein

**Affiliations:** ^1^Ulm University, Institute of Pathology, 89081 Ulm, Germany; ^2^ Ulm University, Institute for Forensic Medicine, 89081 Ulm, Germany

**Keywords:** telomere length, T/C-FISH, alternative pathway

## Abstract

Telomeres are repetitive DNA sequences at chromosomal ends contributing to genomic integrity. In somatic cells, telomeres are shortened during DNA reduplication. Thus, telomere erosion has been regarded as a biological clock. Applying the telomere/centromere (T/C)-FISH technique to human peripheral blood lymphocytes, we showed that pangenomically, telomere shortening is linear in centenarians and that this attrition is delayed in females. Statistics reveal a greater skewness in telomere length distribution in females. As the morphological correlate, we find abnormally long telomeres distributed at random. This "erratic extensive elongation" (EEE) of telomeres is a hitherto unrecognized phenomenon in non-neoplastic cells, and females are more successful in this respect. As evidenced by endoreduplication, EEE is transmitted to the cells' progeny. The mechanism involved is likely to be the alternative pathway of telomere elongation (ALT), counteracting erosion and already known to operate in neoplastic cells.

## Introduction

Even disregarding traumatic deaths due to violence and high-risk lifestyle [1], men have a shorter life span than women. In spite of a multitude of theories, the primary cause is unknown.

Hayflick's hypothesis, that each cell has its own limited division capacity [2], received fundamental support with the discovery of telomeric shortening during DNA replication in somatic cells [3]. In peripheral blood lymphocytes (PBL), a telomeric loss per year of 31 bp (between 2-95 years) [4], 41 bp (between 0-107 years) [5], and 60-66 bp (between 0 and 100 years) [6] is described. Thus, telomere erosion has been regarded as a biological clock (for review see [7]). Whether this also applies to aging at the organismic level is unclear.

**Figure 1.**
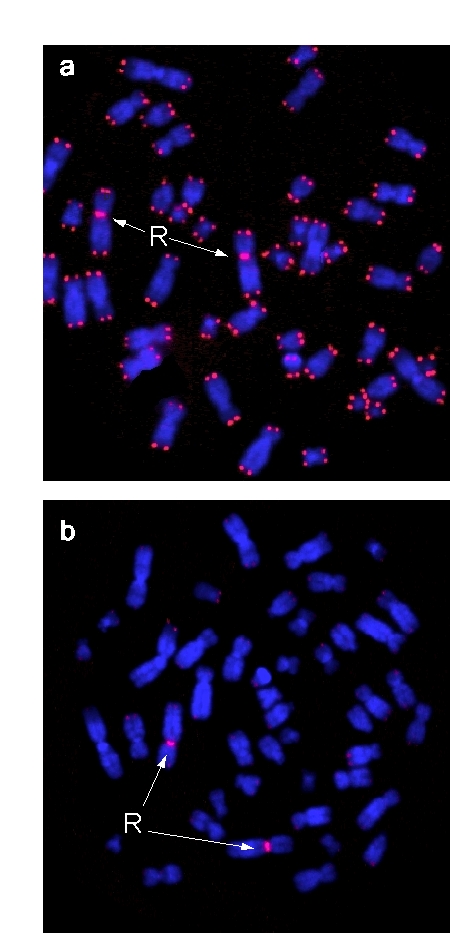
Metaphases of phytohemagglutinin-stimulated peripheral blood lymphocytes. Chromosomes were co-hybridized with peptide nucleic acid (PNA) probes (red) for telomeric sequences and the chromosome 2 centromere serving as internal reference probe, and stained with DAPI (blue). Centromere signals are marked by arrows. (**a**) Metaphase derived from cord blood of a male newborn, (**b**) Metaphase from blood of a centenarian male.

There is,  however, circumstantial  evidence that  this might be the case, as a prospective study showed that death due to cardiac und infectious diseases occurred earlier in those with relatively short telomeres [8]. In Mayer et al. [6], an age-dependent and gender-specific difference in average telomere lengths emerged, women being endowed with longer telomeres. We based our study on PBL from a total of 205 healthy persons, ranging from newborns up to the age of 100 years, and applied T/C- FISH [9] to quantify the telomere lengths of every single chromosome arm (Figure 1).

## Results and discussion

### Telomere erosion is faster in males

Confirming our previous results and that of others [4,8,10,11], we found an age-dependent decline in pan-genomic telomere lengths: this decline proved greater in males [6,9]. To date, the mechanism for this gender difference has proved to be elusive.

### Skewed distribution of telomere length

**Figure 2.**
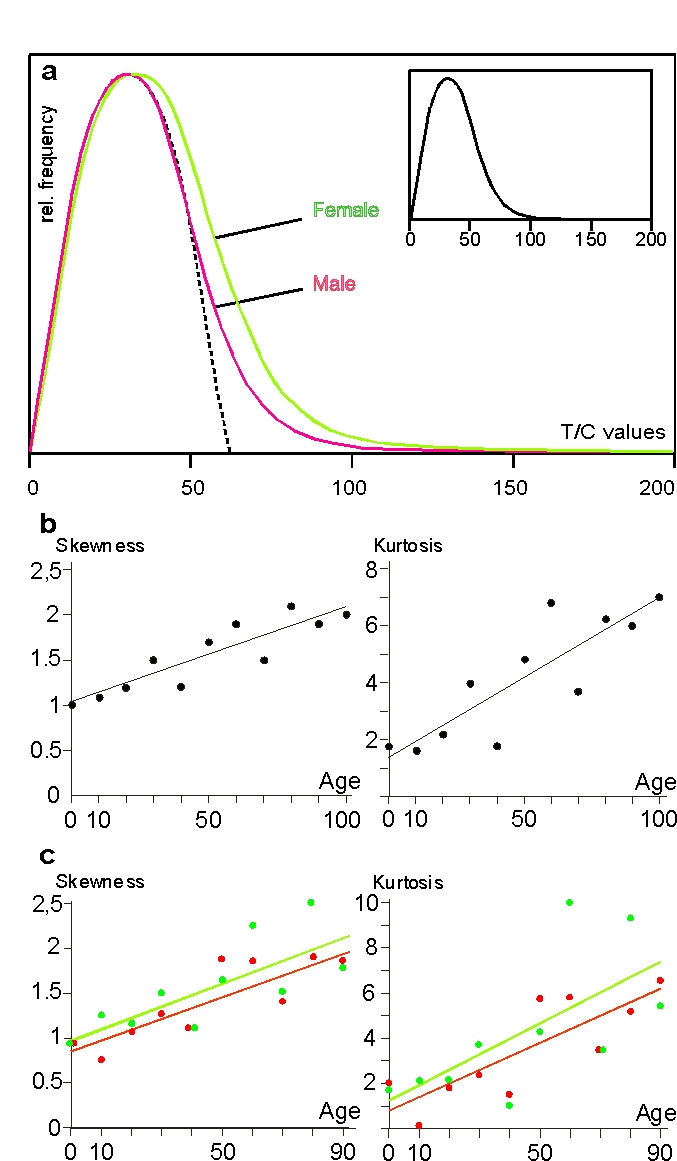
Erratic extensive elongation (EEE) of single telomeres in peripheral lymphocytes. (**a**) Histogram of telomere lengths of all p and q arms of chromosomes of females (green) and males (red). The dotted line represents the theoretical Gaussian distribution, the histogram in the smaller insert represents the curve of female and male values together. The actual curves are skewed to the right. (**b**) Skewness and kurtosis of telomere lengths of p and q arms of chromosomes of lymphocytes in all age groups. Values are limited to 150 T/C values. (**c**) Skewness and kurtosis of telomere lengths of p and q arms of chromosomes of female (filled circles and green line) and male probands (open circles and red line) ranging from newborns up to 90 years. Centenarians are excluded because the male group consists of only 2 persons. Values limited to 150 T/C values as mentioned above. (see “Statistical analysis” for further details).

When we plotted the frequency of a total of 235,266 single telomere values, the resulting curve was not Gaussian but was skewed to the right (Figure 2a, small insert). This is also shown mathematically by ascending skewness and kurtosis, the equation of the regression line of skewness being y=0.0106x+1.0359 with a regression coefficient of r=0.88 and that of kurtosis y=0.0559x+1.3705 (r=0.85) (Figure 2b). Since skew-ness characterizes the degree of asymmetry of a distri-bution around its mean, while kurtosis is a measure of the relative peakedness/flatness of a distribution, our ascending regression lines in Figure 2b indicate that the distribution of telomere lengths becomes more skewed to the right and, at the same time, more peaked with higher age.

### Greater skewness and kurtosis in females

A very obvious new fact emerged when we plotted all our data classified by sex. The skewness of the female distribution curve (1.322) resulting from 115,754 values was greater in extent than the male equivalent (1.099) resulting from 119,472 values (Figure 2a). The difference is significant at a level of a = 0.0098. This is all the more astonishing since the left sides of the curves are almost perfectly superimposed, meaning that the above sex-specific differences in telomere size are not due to differences in frequency of short telomeres but to higher frequencies of long ones in females. Figure 2c shows skewness and kurtosis classified by sex with skewness equations of y=0.013x+0.98 (r=0.78) for females and y=0.012x+0.85 (r=0.86) for males and the kurtosis equations of y=0.07x+1.25 (r=0.67) for females and y=0.06x+0.79 (r=0.82) for males. In both criteria, the female line runs above the male line, corroborating the histograms in Figure 2a and both lines run nearly parallel, meaning that skewness and kurtosis do not essentially differ between genders during a lifetime.

**Figure 3.**
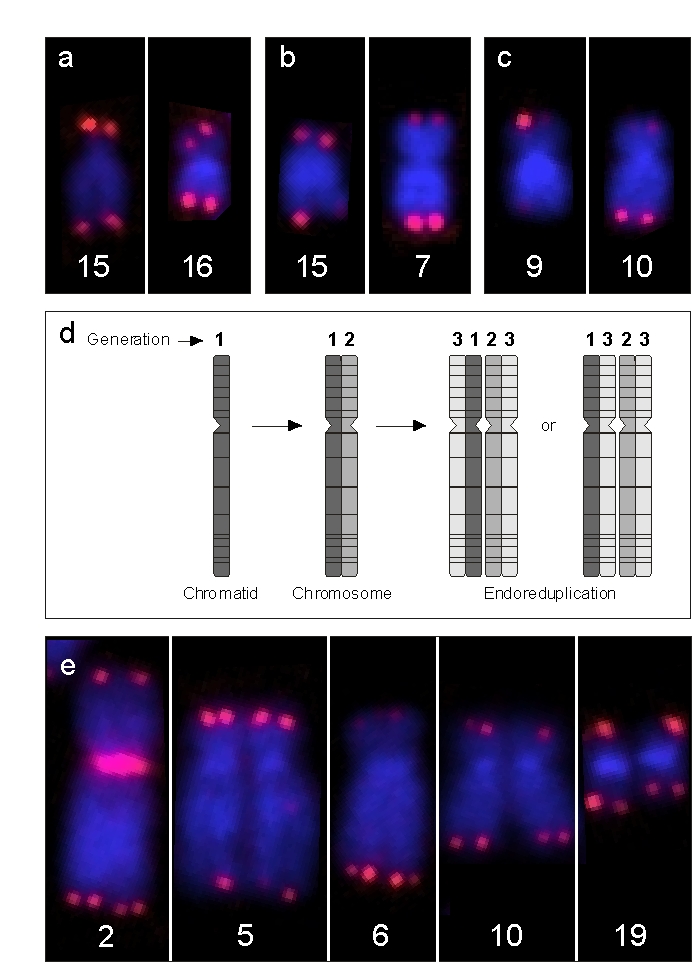
Examples of EEE. (**a**) Depicts chromosomes of a newborn male with enhanced signal intensity at one p arm of chromosome 15 and at both chromatids of chromosome 16, (**b**). This is also the case at a single chromatid of 15q and on both chromatids of chromosome 7q in a 50-year-old male and, (**c**) at a single chromatid of chromosome 9p and at both arms of chromosome 10q in a male centenarian. (**d**) Schematic view of endoreduplication (ER), the result being a group of four homologous chromatids which emerged from one single chromatid. Note that the juxtapositions of the sister and descendant chromatids may be variable, since their three-dimensional packing is broken up by chromosome spreading. (**e**) Single chromosomes of a sporadic ER observed in a 40-year-old female with features of EEE. Since telomeric EEEs at 2p, 5q, 6q, and 10p are doublets and not quadruplets, EEE at these positions must have occurred one cell cycle prior to the ER event. In addition, there is a single telomeric EEE at 19q, which must have occurred during the S-phase directly preceding this ER.

What was the morphologic correlate of the abnormally high T/C FISH medians? Re-examination of our basic data, i.e. the hybridized metaphases, revealed an overall infrequent occurrence of extremely long telomeres of single chromosome arms. This was most evident in the elderly with pangenomically shorter telomeres (Figure 3c). These extremely long, single telomeres appeared to be randomly distributed all over the genome and were singularities, since we could not relocate an identical event in other metaphases of the same individual. These elongations, however, often occurred in doublets, meaning that both chromatids of the same p or q arm were elongated. This strongly suggests that, once the elongation has been achieved in a single chromatid, this newly acquired long telomere is the template for the next generation of descendants of this single individual cell. We coined the term "erratic extensive elongation" (EEE) of single telomeres to define this phenomenon. Once identified, we also found EEE of telomeres in younger people (Figure 3b) and even in newborns, although it was hardly visible and only detectable by means of the software, (Figure 3a). It is conceivable that minor differences in telomere lengths of individual chromosome arms might be the result of EEE that occurred years earlier and was subsequently exposed to general pangenomic erosion. We think it highly likely that EEE is responsible for the skewness of the distribution of telomere values (Figure 2a).

Evidence that EEE is propagated to the cells' progeny came from an anecdotal observation in a middle-aged woman in whose PBL we encountered a considerable number of endoreduplications (ER), together with an increased frequency of EEE. ER is the result of a dysregulation in cell-cycle progression that ends not in normal mitosis but in re-entering of the next cycle, leading to tetraploidy with descendant chromosomes closely attached [12] (Figure 3d). Every "tetrad" of chromatids represents 3 generations. If EEE is detected on two of four telomeres of comparable length, it should be derived from the EEE of an ancestor chromatid. In Figure 3e, this is visible at the telomeres of chromosome 2p, 5p, 6q, and 10p.

A broadly accepted theory explains accelerated telomere shortening by oxidative stress (ROS), with men having the greater load [23-28]. Assuming that ROS causes DNA damage directly, forcing the cell either to repair or to die, the result should be telomere length distributions in two isomorphic curves with a parallel shift of the female curve to the right. As we show, this is not the case. Therefore, the curves we obtained are most likely the result of a mechanism counteracting telomeric loss which is more active/effective in females.

Phenomenologically, EEE is more likely to be a repair of accidental individual telomere damage than a mechanism operating continuously. Following this line of argument, it is very unlikely that telomerase is responsible for EEE, since telomerase-stabilized telomeres show constant lengths of around 4-10 kb in humans (for review, see [13]). A fitting candidate mechanism is the alternative pathway (ALT). ALT is involved in maintenance or lengthening of telomeres, uses enzymes operative in DNA recombination and rep- lication, and can create extreme heterogeneity in telomere lengths [13] by using so-called telomeric circles. Theoretically, these circles can arise from telomeres by means of homologous recombination [14] and might be used by telomerase as a template to elongate telomeres as has been proposed [15].

ALT was found to be active in neoplastic cells and immortalized cell lines with or without telomerase [14]. A physiological role of ALT is suggested by the observation that telomerase-null mice show some degree of telomere elongation in B-lymphocytes during germinal center reaction [16]. Furthermore, Rad54-null mice have significantly shorter telomeres than wild animals, in spite of unimpaired telomerase activity [17]. Rad54 is involved in DNA recombination and may be part of ALT. We frequently found telomeric EEE of around 150 T/C values (corresponding to 33 kb) and, in rare cases, even up to 300 T/C value (63 kb). Our observations thus match data of mean telomere lengths of over 50 kb, as measured by terminal restriction fragment analysis in so-called human ALT-positive cell lines [18]. Finally, if ALT is indeed the key player in EEE, which still has to be proven, the question remains whyfemales use ALT more effectively.

As we and others have clearly demonstrated a linear decline in telomere length [4,6,8-11], it seems at least unlikely that sex hormones play a role. It is, however, well documented that exposure to reactive oxygen species (ROS) leads to damage in nuclear DNA (see [19] and reviews [20,21]), and to telomere shortening [22], and a growing body of evidence indicates that males are subject to higher levels of cellular stress than females [23-28]. A direct influence of the extracellular environment, the biosphere, on telomere length was recently shown in a pair of twins of different gender with blood chimerism [29]: Compared with their "normal" length (i.e. female cells in the woman and male cells in the man), the telomeres within the male lymphocytes in the female twin were 33% longer than those in the male twin. By contrast, comparison of the telomeres within the female lymphocytes in the female with their counterparts in the male revealed that the latter were shortened to 87% of their "normal" length.

Apart from direct damaging influences, ROS can serve as messengers to control other physiological processes not directly involved in ROS defense [20,30,31]. For example, ROS reacts with plasma thiol to form disulfides. This extracellular thiol/disulfide equilibrium has signaling functions on different cell membrane receptors, e.g. EGFR, and regulates poly ADP-ribose polymerase (PARP) [32,33]. EGFR is involved in the expression of hTERT, which controls telomerase activity [34], and PARP is a key DNA repair enzyme and is involved in DNA replication, recombination [35], telomere maintenance (for review, see [36]), and DNA histone modifications and double-strand break repair [37].

Against this background, it is tempting to speculate on an interplay between ROS, the thiol/disulfide system and the control of telomere length. All the more, since telomere erosion can be slowed by N-acetylcysteine, which changes the thiol/disulfide system and inhibits ROS formation [20,38,39]. In conclusion, this study provides evidence that the gender-specific differences in telomere attrition depend on factors leading, in the female, to enhanced repair/recombination processes in the lymphocytes (visible by EEE) and, finally, to longer telomeres (evident by greater skewness and kurtosis of the female histogram). Time will show whether it contributes to the gender differences in life expectancy.

## Materials and methods

Cell sources. Informed written consent was obtained from each participant and from the parents of the newborns and children. Blood samples from a total of 205 donors were used: 108 umbilical cord blood samples from 55 male and 53 female newborns and 97 peripheral blood samples from donors ranging from 10 to 100 years of age (five female and five male, each in every life decade of life), with the exception of male centenarians, of which we had only two.

Cell culture and metaphase preparation. Heparinized blood was cultured as described [9] to obtain lymphocytes which were then phytohemagglutinin-stimulated to obtain metaphases. Twenty metaphases from each individual were analyzed in a Zeiss Axioscope microscope (Jena, Germany) equipped with a CCD camera and linked to the Isis and telomere software (MetaSystems, Altlussheim, Germany).

Peptide nucleic acid (PNA) probes. The PNA probe for telomeric sequences is a ready-to-use probe included in the Telomere PNA FISH Kit/Cy3 (K5326 from DakoCytomation A/S, Glostrup, Denmark). The PNA centromeric probe for chromosome 2 was generated by Dako and is available upon request. The PNA probe for the centromere 2 was added as 1 μl to 10 μl ready-to-use telomere probe solution leading to a final concentration of 2 ng/μl. The hybridization was performed according to the manufacturer's instructions and is described in detail in reference 9.

Statistical analysis of T/C data . Normalized data were derived by calculating the ratio of the absolute telomere intensities and the reference signal intensities of each metaphase (T/C value). For each individual, about 20 metaphases were analyzed. These primary data were compiled by the software, and mean telomere intensities of the p- and q-arms of each chromosome were obtained. These data were used for further statistics.

Firstly, an attempt was made to characterize the statistical distribution of all individual T/C values as a whole. To this end, the T/C data of all 46 chromosome arms from 102 males (n_1_ = 119,472) and 103 females (n_2_ = 115,754) were used. The skewness of the empirical frequency distribution of the individual T/C values was estimated for the male and the female group using standard software (SAS Institute 2000). The skewness estimates amounted to 1.099 in the male group and to 1.322 in the female group. To test these values for a significant difference, a randomization test based on independent random resampling without replacement was performed in the following manner [40-42]: All data were united to a single univariate set consisting of 235,226 T/C values. From this set, 9999 pairs of random samples with sizes n_1_ = 115,754 and n_2_ = 119,472 were drawn independently without replacement. For each pair of samples, the difference D* between the two estimated skewness values was calculated. The resulting 9999 D*-values of the skewness estimates from the virtual random samples with T/C values from both genders, and the difference D between the skewness estimates originating from the two real samples (male and female only), were sorted by size. A result was considered as significant at the 5%-level if D occupied rank 1-250, or rank 9751-10000 in the whole series of difference values. The D-value from the real sample pair, D = 0.223, occupied rank 9951/10000, hence it was considered significant at a level of α= 0.0098.

Furthermore, the functional relationship between telomere size and age was studied for both sexes. The medians of the T/C values were determined in the 205 individuals separately for the telomeres of all 46 chromosome regions. This part of the study yielded 4896 median T/C values in the male group and 4738 median T/C values in the female group. Linear regression analysis led to slope estimates ofg = -0.302 for the female group and g = -0.328 in the male group, i.e. the estimated regression line showed negative gradients with a slightly steeper descent for the male gender. A randomization test with resampling without replacement, analogous to the approach described above, was performed to test the slopes of the two regression lines for a significant difference. To this end, the two bivariate samples were united to a single bivariate sample with 9634 elements. From this united sample, 9999 pairs of bivariate samples (with age and T/C values from both genders) of sizes n_1 _= 4896 and n_2_ = 4738 were drawn at random without replacement. For both virtual samples of each pair, regression lines of T/C value on age were fitted by standard methods. The difference D* between the two slope estimates was recorded for each virtual sample pair. Thereafter, the 999 D*-values and the difference value D = 0.026 from the pair of regression lines corresponding to the true female and male sample were ranked by size. The true D-value occupied rank 10000/10000 in this series, hence it was considered significant at a level of α ≤ 0.0001.
